# All-Cause and Cause-Specific Mortality in Children With Congenital Zika Syndrome in Brazil

**DOI:** 10.1001/jamanetworkopen.2024.56042

**Published:** 2025-01-23

**Authors:** Luciana Lobato Cardim, Maria da Conceição Nascimento Costa, Laura Cunha Rodrigues, Rita Carvalho-Sauer, Elizabeth Bailey Brickley, Ricardo Arraes de Alencar Ximenes, Julia Moreira Pescarini, Roberto Fernandes Silva Andrade, Mauricio Lima Barreto, Maria da Glória Lima Cruz Teixeira, Enny Santos da Paixao

**Affiliations:** 1 Center of Data and Knowledge Integration for Health, Gonçalo Moniz Institute, Oswaldo Cruz Foundation, Salvador, Bahia, Brazil; 2 Collective Health Institute, Federal University of Bahia, Salvador, Bahia, Brazil; 3 London School of Hygiene and Tropical Medicine, London, United Kingdom; 4 East Regional Health Center, State Health Secretariat of Bahia, Santo Antonio de Jesus, Bahia, Brazil; 5 Federal University of Pernambuco, Recife, Pernambuco, Brazil; 6 Physics Institute, Federal University of Bahia, Salvador, Bahia, Brazil

## Abstract

**Importance:**

Congenital Zika syndrome (CZS) can lead to a range of developmental and neurological issues, which increases the risk of early death. However, the all-cause and cause-specific mortality in children with CZS in the first 5 years of life remain unknown.

**Objective:**

To compare the hazard of all-cause and cause-specific mortality before age 5 years among children with and without CZS in Brazil.

**Design, Setting, and Participants:**

This cohort study used nationwide linked routine data including all children born from January 2015 to December 2018 in Brazil. They were followed-up until age 5 years, death, or December 2020, whichever occurred first. All analysis were conducted in May 2024.

**Exposures:**

Confirmed or probable cases of CZS.

**Main Outcomes and Measures:**

All-cause and cause-specific deaths from respiratory, infectious and parasitic, and nervous system diseases were the outcomes. Hazard ratios (HRs), comparing children with and without CZS, for all and cause-specific mortality were estimated using Cox proportional hazard models adjusted for region and year of birth, maternal characteristics (age, education, race and/or ethnicity, and marital status), and sex of the newborn. The analysis was conducted in May 2024.

**Results:**

In total, there were 11 387 431 live births (5 832 594 male newborns [51.2%]). Of 3080 children notified as CZS cases, 444 (14.4%) died, including 154 (34.7%) from respiratory diseases, 152 (34.2%) from infectious and parasitic diseases, and 82 (18.5%) from nervous system diseases. Children with CZS were predominantly preterm (596 newborns [20.0%] vs 1 122 378 newborns [10.1%]) and had low birth weight (1095 newborns [35.7%] vs 805 373 newborns [7.1%]), compared with children without CZS. After controlling for confounders, children with CZS were 13.10 (95% CI, 11.86-14.46) times more likely to die in the first 5 years of life compared with those without the syndrome. The cause-specific mortality HRs were 30.28 (95% CI, 25.59-35.83) for respiratory diseases, 28.26 (95% CI, 23.85-33.48) for infectious and parasitic diseases, and 57.11 (95% CI, 45.23-72.11) for nervous system diseases. After excluding newborns who were preterm, low birth weight, and/or small for gestational age, the HRs for all-cause and cause-specific deaths were even higher.

**Conclusions and Relevance:**

In this cohort study, children born with CZS had strikingly higher risk of overall and cause-specific mortality. These findings may support the development of clinical protocols to prevent early mortality and improve survival in these children.

## Key Points

**Question:**

Among children with and without congenital Zika syndrome (CZS) in Brazil, what are the overall and cause-specific (respiratory, infectious and parasitic, and nervous system diseases) mortality risks during the first 5 years of life?

**Findings:**

In this population-based cohort study of nearly 11.4 million live births, children younger than 5 years born with CZS had a 13.10-fold higher hazard of death compared with those without CZS. The cause-specific mortality hazard ratios were 30.28 for respiratory, 28.26 for infectious and parasitic, and 57.11 for nervous system diseases.

**Meaning:**

These findings might help develop protocols to prevent early mortality and improve survival in children with CZS.

## Teaser

This cohort study examines the hazards of all-cause and cause-specific mortality among children younger than 5 years with and without congenital Zika syndrome (CZS) in Brazil.

## Introduction

Zika virus (ZIKV) can be vertically transmitted during pregnancy^1^ and causes adverse fetal outcomes, including severe malformations of the central nervous system^2^ and disabilities arising from central nervous system damage.^3,4^ Previous research^5^ in Brazil suggests that children born with congenital Zika syndrome (CZS) have 11 times higher mortality in the first 3 years of life compared with peers born without CZS. The most frequent causes of death among these children were respiratory, infectious and parasitic, and nervous systems diseases.^5,6^

Children with severe CZS are at high risk of developing dysphagia,^7^ which may increase risks of aspiration pneumonia leading to respiratory failure.^8^ A cohort study^9^ in Brazil found that a high proportion of children with ZIKV-related microcephaly develop early epilepsy with poor seizure control, which may increase their mortality risk. Furthermore, children with CZS have increased risks of developing hydrocephalus,^10^ which can cause death from nervous system diseases. Preliminary evidence suggests that children with CZS may have altered immune phenotypes^11,12^ and low reactivity to tuberculin skin test following receipt of the BCG vaccine.^11^ Although research remains limited, it has been hypothesized that these immune differences may increase risks of developing severe infectious diseases in children with CZS.

As the children born in Brazil during the ZIKV epidemic get older, data have now become available for investigating mortality up to 5 years. This study aims to estimate the relative risks of overall and cause-specific mortality from respiratory, infectious and parasitic, and nervous system diseases in the first 5 years in children with and without CZS in Brazil.

## Methods

### Study Design

We conducted a longitudinal, population-based cohort study, linking administrative data reporting live births, notified cases of CZS, and deaths in Brazil. We included births from January 1, 2015, to December 31, 2018, and deaths from January 1, 2015, to December 31, 2020. The follow-up period began at birth and lasted until December 31, 2020, death, or the age of 5 years. We followed the Strengthening the Reporting of Observational Studies in Epidemiology (STROBE) reporting guidelines. This study was approved by the Research Ethics Committee of the Federal University of Bahia, Institute of Health Collective. Informed consent was waived, because the data were deidentified and analyzed under strict security procedures, in accordance with the General Data Protection Law (13,709/2018), Article 7, Item IV.

### Data Source

#### Births

The Live Births Information System (SINASC) contains data from all live births in Brazil. In the study period, SINASC average coverage was 97%.^13^ From SINASC, we extracted information on the date and region of birth, infant sex, birth weight, congenital abnormalities, and maternal characteristics (age, education, race and ethnicity, marital status, gestational age at birth, and number of fetuses). In Brazil, maternal race (skin color) is self-reported and categorized into 5 groups: Black, Indigenous, *Pardo* (ie, multiracial), White, and Yellow. This classification was established in 1940 and has remained unchanged; however, to avoid offensive connotations, we refer to the Yellow category as *Asian descent* in this article. Race and ethnicity serve as proxies for health and social inequalities in the Brazilian context.^14^

#### Notification of CZS

The Public Health Event Record (RESP) is an online form developed by the Brazilian Ministry of Health with the purpose of recording cases during public health emergencies.^15^ In Brazil, fetuses and live births who meet criteria for suspected CZS^16^ must be reported to RESP (eMethods in Supplement 1). After notification, all suspected cases of CZS are investigated by epidemiological surveillance teams and classified as confirmed, probable, inconclusive, or ruled out. From RESP, we extracted information on the final classification of the CZS cases and on notifications of other STORCH (syphilis, toxoplasmosis, rubella, cytomegalovirus, and herpes simplex) congenital infections.

#### Deaths

The Mortality Information System (SIM) contains data on all deaths in Brazil. In the study period, SIM average coverage was 96%.^13^ From SIM, we retained information on the date and cause of death by *International Statistical Classification of Diseases and Related Health Problems, Tenth Revision (ICD-10)* code.

### Linkage Process

Live births records from SINASC were linked to records from RESP and SIM. The linkage was performed using a nondeterministic record linkage tool (CIDACS-RL), developed to conduct large-scale administrative data linkage from Brazil.^17^ The accuracy of each linkage was assessed by manually verifying a random sample of records and evaluating sensitivity and specificity using receiver operating characteristic curves. For the linkage of SINASC to RESP, the sensitivity was 96%, and the specificity was 95%. For the linkage of SINASC to SIM, the sensitivity varied from 88% in 2019 to 95% in 2017, and specificity varied from 89% in 2020 to 98% in 2017. Linkage procedures were conducted at the Center of Data and Knowledge Integration for Health at Oswaldo Cruz Foundation in a strict data protection environment and according to ethical and legal rules.^18^ After linkage, the data were deidentified and provided to the researchers for analysis in a safe haven.

### Procedures

All singleton live births in Brazil were eligible for this study. We selected births from January 2015 onward, as ZIKV infections were laboratory-confirmed in Brazil in May 2015, with evidence suggesting possible earlier circulation.^19^ We excluded live births with other congenital abnormalities registered in SINASC, other congenital STORCH infections registered in RESP, and/or an inconclusive, under investigation, or ruled out classification of CZS in RESP.

The outcomes were all-cause and cause-specific deaths in children younger than 5 years. All-cause death was defined on the basis of live births that could be linked to records in SIM, regardless of the cause of death. Cause-specific deaths were categorized as follows: deaths from respiratory system diseases (*ICD-10* codes J00-J99), infectious and parasitic diseases (*ICD-10* codes A00-B99), and nervous system diseases (*ICD-10* codes G00-G99), all linked to SIM. The specific causes were considered to be an outcome if they were registered in any of the causes of death lines from the death certificate.

**Figure 1.**
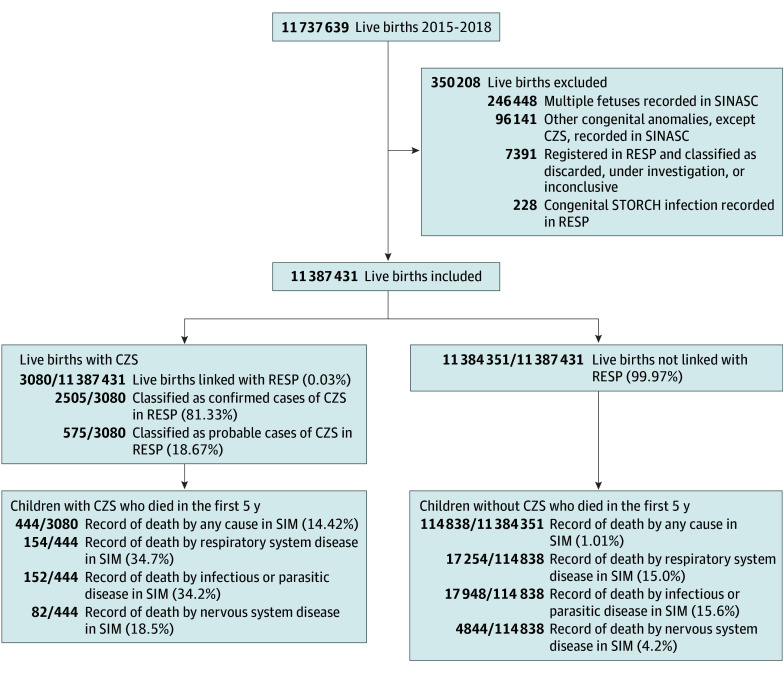
Flowchart of Study Population of Live Births in Brazil, 2015 to 2018 CZS indicates congenital Zika syndrome; RESP, Public Health Event Record; SIM, Mortality Information System; SINASC, Live Birth Information System; and STORCH, Syphilis, Toxoplasmosis, Rubella, Cytomegalovirus, Herpes Simplex Virus.

The exposure CZS cases were defined as all live births linked to a record from RESP classified as confirmed or probable CZS cases (Figure 1). Low birth weight was defined as less than 2500 g, and preterm birth was defined as birth occurring before 37 weeks of gestation. Small for gestational age children were live born below the 10th weight percentile for gestational age and sex, according to the INTERGROWTH-21st standards.^20^

### Statistical Analysis

Descriptive statistics were presented for maternal sociodemographic data and newborn characteristics for children with and without CZS. This study includes all live births in the country, resulting in a very large sample size. For this reason, we did not report *P* values, as they can become extremely small owing to the large sample size, often indicating statistically significant results that may not reflect meaningful differences.

All-cause and cause-specific mortality rates were estimated as deaths per 10 000 person-years (PY). Crude hazard ratios (HRs) with 95% CIs comparing live births with and without CZS were estimated using Cox proportional hazards models. In the adjusted model, we included the region and year of birth, maternal characteristics (age, education, race and ethnicity, and marital status), and sex of the newborn. For the adjusted model, we conducted a complete-case analysis. The baseline characteristics of live births included and excluded from the adjusted analysis are available in eTable in Supplement 1. We estimated mortality up to age 5 years and conducted specific models by age group (<1 year, 1 to <2 years, 2 to <3 years, 3 to <4 years, and 4 to <5 years). In this analysis, we fitted regular Cox model for each age group. Follow-up began at birth or the specified age, and once a child reached the upper limit of an age group, they were censored.

Sensitivity analysis was also performed with the cohort restricted to children born with a gestational age at birth greater than or equal to 37 weeks, a birth weight greater than or equal to 2500 g, and/or adequate size for gestational age. This analysis enabled us to estimate the HRs of all-cause and cause-specific mortality independently of preterm delivery, low birth weight, and small for gestational age. All analyses were conducted in May 2024.

## Results

During the study period, 11 737 639 live births were recorded in Brazil, 11 387 431 (97.0%) of which were included in this study (5 832 594 male newborns [51.2%]). Of those, 3080 children (0.03%) were classified as having confirmed or probable cases of CZS. When we compared maternal characteristics of children born with and without CZS, mothers of children with CZS were more likely to identify as being Black or *Pardo* race or ethnicity (2328 parents [80.8%] vs 6 782 521 parents [62.4%]), to be younger (age <20 years, 712 parents [23.1%] vs 1 943 408 parents [17.1%]), to have less education (≥8 years of education, 2245 parents [73.9%] vs 8 985 119 parents [80.1%]) and single (1557 parents [51.2%] vs 4 852 943 parents [43.1%]). Children with CZS were predominantly female (1630 newborns [53.1%] vs 5 552 572 newborns [48.8%]), born in the Northeast region (1915 newborns [62.2%] vs 3 203 191 newborns [28.1%]), preterm (596 newborns [20.0%] vs 1 122 378 newborns [10.1%]), had low birth weight (1095 newborns [35.7%] vs 805 373 newborns [7.1%]), and were small for gestational age (1065 newborns [36.3%] vs 785 264 newborns [7.1%]) compared with children without CZS (Table).

**Table.** Baseline Characteristics Live Births in the Cohort Linked Data by CZS Status

Characteristics	Live births, No. (%) (N = 11 387 431)	
	Without CZS (n = 11 384 351)	With CZS (n = 3080)
Maternal age, y		
<20	1 943 408 (17.1)	712 (23.1)
20-34	7 862 653 (69.1)	2034 (66.0)
≥35	1 578 290 (13.9)	334 (10.8)
Missing	0	0
Maternal education, y		
None	54 466 (0.5)	18 (0.6)
1-3	262 338 (2.3)	95 (3.1)
4-7	1 917 371 (17.1)	679 (22.4)
≥8	8 985 119 (80.1)	2245 (73.9)
Missing	165 057 (1.5)	43 (1.4)
Maternal race or ethnicity		
Asian descent	44 259 (0.4)	11 (0.4)
Black	604 464 (5.6)	191 (6.6)
Indigenous	93 001 (0.9)	19 (0.7)
* Pardo*	6 178 057 (56.8)	2137 (74.2)
White	3 952 293 (36.4)	524 (18.2)
Missing	512 277 (4.5)	198 (6.4)
Marital status		
Single	4 852 943 (43.1)	1557 (51.2)
Widow	19 537 (0.2)	5 (0.2)
Divorced	132 986 (1.2)	25 (0.8)
Married or civil union	6 251 746 (55.5)	1454 (47.8)
Missing	127 139 (1.1)	39 (1.3)
Year of birth		
2015	2 923 638 (25.7)	1174 (38.1)
2016	2 770 908 (24.3)	1436 (46.6)
2017	2 834 055 (24.9)	309 (10.0)
2018	2 855 750 (25.1)	161 (5.2)
Birth region		
Southeast	4 466 729 (39.2)	707 (23.0)
North	1 227 933 (10.8)	170 (5.5)
Northeast	3 203 191 (28.1)	1915 (62.2)
South	1 541 770 (13.5)	42 (1.4)
Central West	944 728 (8.3)	246 (8.0)
Missing	0	0
Sex of the newborn		
Female	5 552 572 (48.8)	1630 (53.1)
Male	5 831 152 (51.2)	1442 (46.9)
Missing	627 (<0.1)	8 (0.3)
Gestational age at birth, wk		
<32	150 568 (1.4)	97 (3.3)
32-36	971 810 (8.7)	499 (16.7)
≥37	10 034 050 (89.9)	2386 (80.0)
Missing	227 923 (2.0)	98 (3.2)
Birth weight, g		
500-1499	115 402 (1.0)	140 (4.6)
1500-2499	689 971 (6.1)	955 (31.1)
2500-7000	10 561 005 (92.9)	1979 (64.4)
Missing	17 973 (0.2)	6 (0.2)
Small for gestational age		
Yes	785 264 (7.1)	1065 (36.3)
No	10 242 025 (92.9)	1868 (63.7)
Missing	357 062 (3.1)	147 (4.8)

Abbreviation: CZS, congenital Zika syndrome.

Of 3080 children with CZS, 444 (14.4%) died during the first 5 years of life. Among these, 154 (34.7%) died from respiratory diseases, 152 (34.2%) from infectious and parasitic diseases and, 82 (18.5%) from nervous system diseases. Of the 11 384 351 newborns without CZS, 114 838 (1.0%) died. Among these, 17 254 (15.0%) died from respiratory diseases, 17 948 (15.6%) from infectious and parasitic diseases and, 4844 (4.2%) from nervous system diseases (Figure 1).

**Figure 2.**
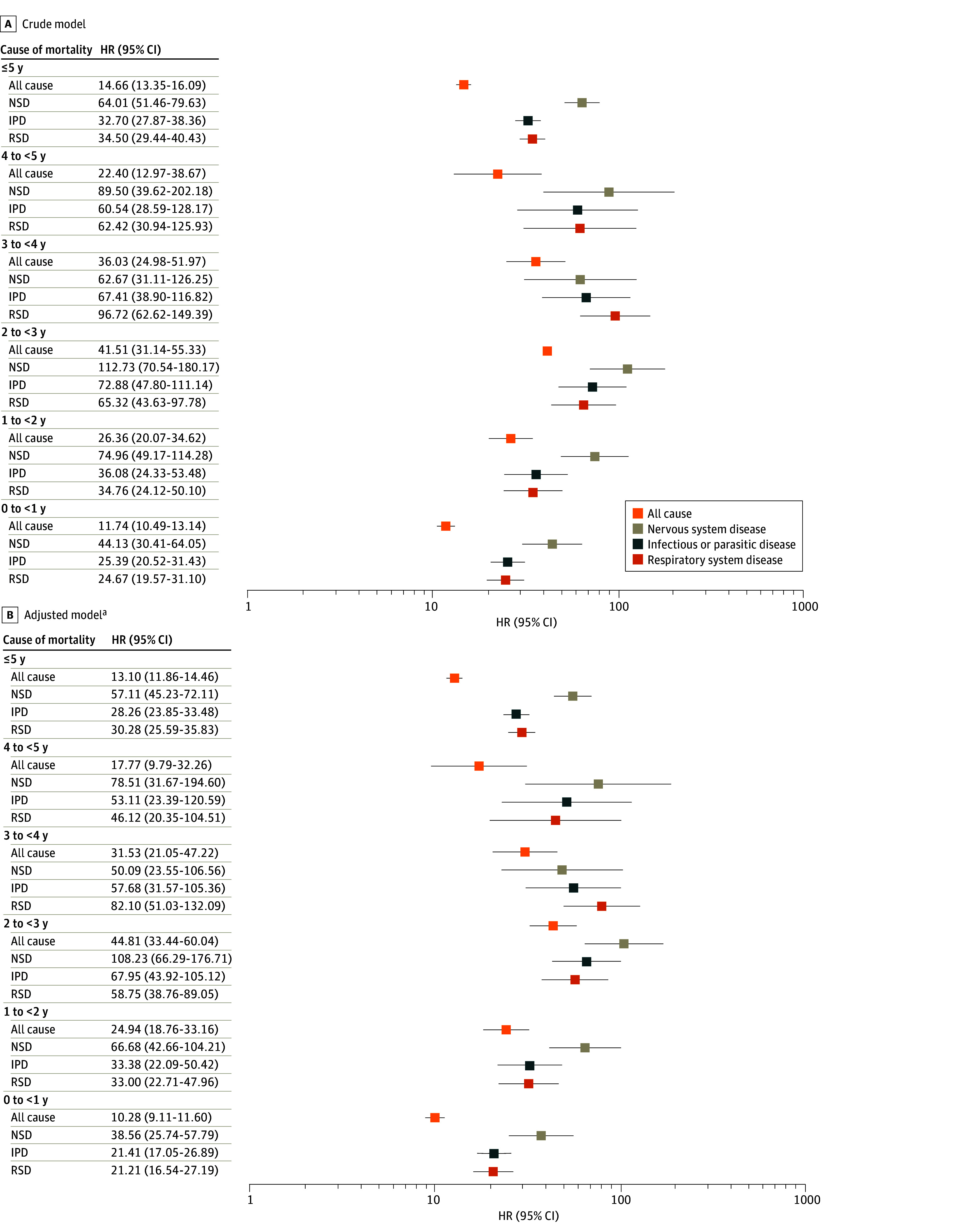
All-Cause and Cause-Specific Mortality Hazard Ratios (HRs) From Respiratory System Disease (RSD), Infectious and Parasitic Disease (IPD), and Nervous System Disease (NSD), by Age Group, of 11 387 431 Live Births in the Cohort Linked Data, Brazil, 2015-2018 ^a^Adusted by region, year of birth, maternal age, maternal education, maternal race or ethnicity, marital status, and sex of the newborn.

The all-cause mortality rate in the first 5 years of life was 416.42 deaths per 10 000 PY (95% CI, 379.44-457.02 deaths per 10 000 PY) among children with CZS and 29.08 deaths per 10 000 PY (95% CI, 28.91-29.25 deaths per 10 000 PY) among children without CZS. In the adjusted model, children with CZS had a 13.10-fold higher hazard of death (95% CI, 11.86-14.46) compared with children without CZS. The cause-specific mortality HRs over the 5 years comparing the children with and without CZS were 30.28 (95% CI, 25.59-35.83) for respiratory system diseases, 28.26 (95% CI, 23.85-33.48) for infectious and parasitic diseases, and 57.11 (95% CI, 45.23-72.11) for nervous system diseases (Figure 2).

**Figure 3.**
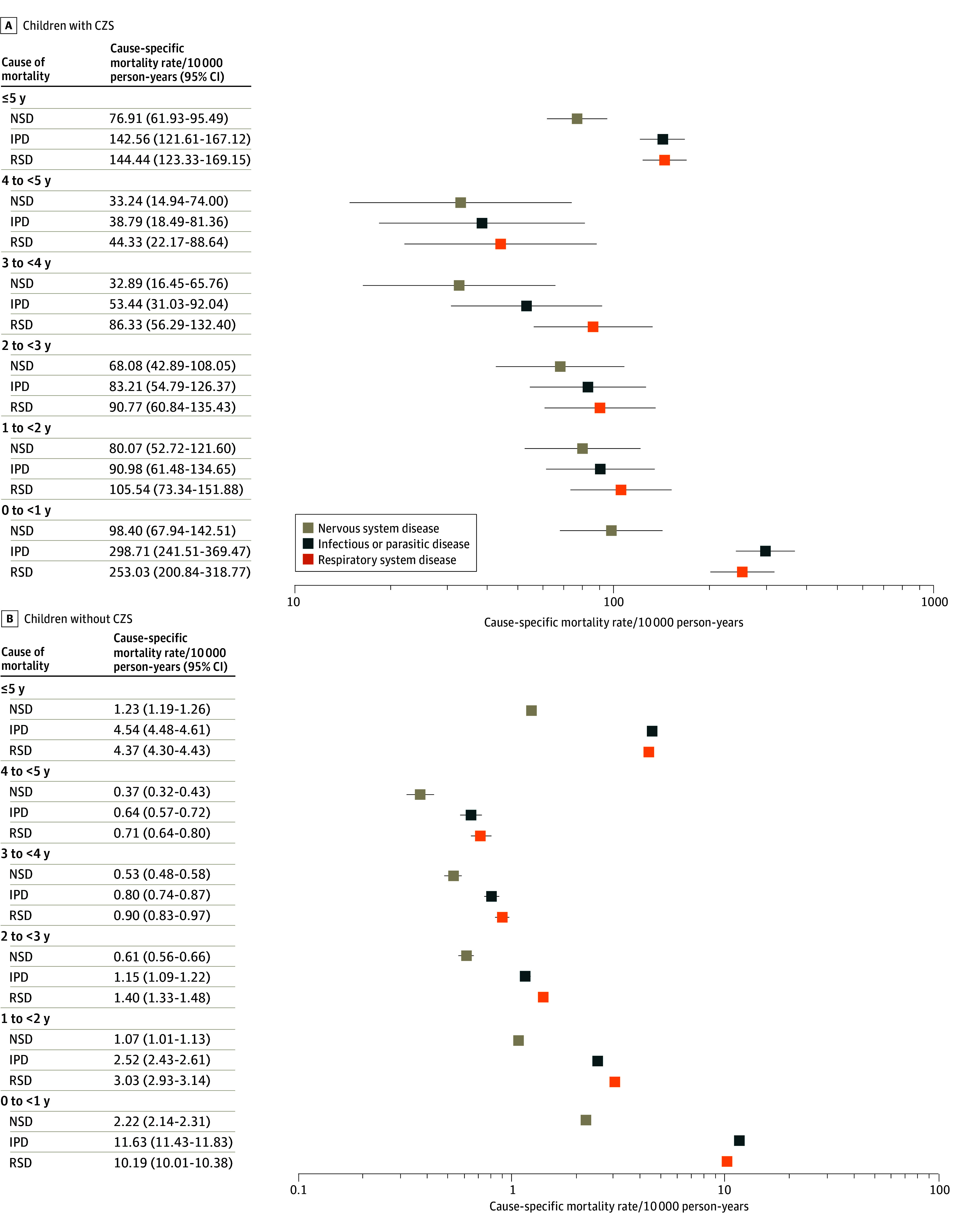
Cause-Specific Mortality Rate, Per 10 000 Person-Years, by Age Group, of 11 387 431 Live Births in the Cohort Linked Data, Brazil, 2015-2018 CZS indicates congenital Zika syndrome; IPD, infectious and parasitic diseases; NSD, nervous system diseases; and RSD, respiratory system diseases.

In the analyses by age group, we found that the highest cause-specific mortality rates per 10000 PY were observed in infants younger than 1 year for both children with and without CZS. For children with CZS vs those without CZS, mortality rates were 253.03 deaths per 10 000 PY vs 10.19 deaths per 10 000 PY for respiratory system diseases, 298.71 deaths per 10 000 PY vs 11.63 deaths per 10 000 PY for infectious and parasitic diseases, and 98.40 deaths per 10 000 PY vs 2.22 deaths per 10 000 PY for nervous system diseases (Figure 3).

The risk of death was highest at the age of 2 years for all causes of death (HR, 44.81; 95% CI, 33.44-60.04), infectious and parasitic diseases (HR, 67.95; 95% CI, 43.92-105.12), and nervous system diseases (HR, 108.23; 95% CI, 66.29-176.71). For respiratory diseases, the risk peaked at age 3 years (HR, 82.10; 95% CI, 51.03-132.09) (Figure 2).

When we restricted the analysis to 9 488 463 live births born at full term, weighing 2500 g or more, and with an adequate size for gestational age, we observed that the mortality rate of children with CZS was also higher compared with those without CZS. In the adjusted model, children younger than 5 years with CZS had a 16.94 times higher hazard of death (95% CI, 14.14-20.30) compared with those without CZS. Cause-specific mortality HRs comparing children with and without CZS were 28.03 (95% CI, 21.09-37.25) for respiratory disease, 29.64 (95% CI, 22.16-39.64) for infectious and parasitic diseases, and 69.77 (95% CI, 49.63-98.09) for nervous system diseases (eFigure in Supplement 1).

## Discussion

In this population-based cohort study following up approximately 11.4 million live births in Brazil, we found evidence that children with CZS had strikingly higher rates of death overall and from the specific causes of respiratory, infectious and parasitic, and nervous system diseases than those without the syndrome. Although the all-cause and cause-specific mortality rates generally decreased after the first year of life, the mortality HRs comparing children with and without CZS tended to increase with age. Children with CZS at 2 years of age had approximately 45 times greater risk of death from all causes, approximately 70 times greater risk of death from infectious and parasitic diseases, and more than 100 times greater risk of death from nervous system diseases, compared with children without CZS at the same age in adjusted models. Children with CZS at 3 years of age had approximately 80 times greater risk of death from respiratory system diseases compared with their counterparts without CZS. These patterns persisted even when excluding preterm and/or small newborns from the analyses, suggesting that the increased risk of death in children with CZS cannot be solely attributed by those adverse birth outcomes.

Children with CZS have severe structural anomalies and functional disabilities that can contribute to their increased mortality HR compared with their counterparts. In children with CZS, dysphagia may favor the premature escape of the bolus into the pharynx, increasing the risk of death due to airway obstruction and the acquisition of respiratory infections caused by bronchoaspiration.^8,21^ This could potentially explain the high respiratory system mortality risk. Furthermore, other factors in children with CZS, such as phrenic nerve palsy with diaphragmatic paralysis^22^ and seizures,^23^ can increase the risk of pulmonary aspiration, leading to respiratory failure.

Because of the severity of brain lesions, epilepsy is a common finding and frequent cause of increased mortality in children with CZS.^24^ In a cohort study^9^ performed in Brazil, the cumulative incidence of epilepsy in children with ZIKV-related microcephaly was 71% in the first 2 years of life, and the overall response rate to antiepileptic treatment was considered low. These factors may have contributed to the high risk of death from nervous system diseases in children with CZS. Children with CZS may experience a progressive decrease in brain mass size while cerebrospinal fluid production remains active. Together, these mechanisms can lead to severe hydrocephalus and intracranial hypertension, increasing the risk of death.^10^

Through monitoring the first generation of children affected by CZS, many others disorders are being discovered. In a case-control study,^11^ it was observed that children with CZS have deficient cellular immunological memory, demonstrated by low reactivity to the tuberculin skin test in children who received the BCG vaccine less than 2 years ago. Further research will be valuable for understanding the extent of the immune differences in children with CZS and how they may influence the risk of death from infectious and parasitic diseases.

We observed that CZS was more common among children of socially vulnerable women. Social factors are associated with increased child mortality and difficulties in accessing health care services.^25^ Families of children with CZS have demands for specialized care and encounter a multitude of inequities and barriers when trying to access this care.^26^ Thus, the complexity in managing the various social adversity linked with CZS may contribute to the higher risk of early mortality among these children.

A strength of our study was the large sample size, including all confirmed and probable CZS cases registered in Brazil. We also included a population-representative comparison group. Since we had access to birth information, we conducted sensitivity analyses excluding infants with low birth weights, preterm births, and births that were small for gestational age.

### Limitations

Our study also has limitations. It was based on administrative registry data, which may be incomplete. There is underreporting in RESP, mainly among those fetuses prenatally exposed to ZIKV but without detectable malformations immediately after birth, with overrepresentation of microcephalic cases, since this was the main criterion for registration in the system at the beginning of the epidemic, which can lead to an overestimation of the risk, because the full spectrum CZS may not be represented in the notified cases. The absolute risk for microcephaly at birth or first evaluation in women infected with Zika during pregnancy was 2.6%, and that for microcephaly detected at any time during follow-up from 13 ZIKV-infected pregnancy cohorts in Brazil was 4.0%.^2^ The prevalence of asymptomatic ZIKV infections in the general population after Zika epidemics is high; a systematic review^27^ found that the prevalence ranged from 29% in French Polynesia to 82% in Yap State. This may have led to an underestimation of the difference between the 2 groups, as measured by the HR. Also, there may be underregistration the cause of death as from respiratory, infectious and parasitic, and nervous system diseases leading to an underestimation of the risk. The linkage process may have introduced classification bias due to linkage errors; however, we believe this would lead to nondifferential misclassification.

## Conclusions

In this cohort study of 11.4 million live births, children with CZS showed a high vulnerability to all-cause and cause-specific mortality up to 5 years of age. These findings may aid in developing protocols to prevent early mortality and improve survival in these children. In addition, the results underscore the importance of active surveillance for ZIKV transmission, the need for vaccine and antiviral development to reduce vertical transmission, and adequate care for these children, especially during and after infectious disease epidemics.

## Notes

Supplement 1. **eMethods**. Criteria for defining a suspected case of Congenital Zika Syndrome (CZS), defined by the Brazilian Ministry of Health
eReferences
**eTable.** Baseline characteristics of live births included and excluded from the adjusted analysis, according to congenital Zika syndrome (CZS) Status, Brazil, 2015-2018 **eFigure.** All-cause and cause-specific mortality hazard ratio from respiratory, infectious and parasitic, and nervous system diseases, by age group, of 9,488,463 live births born at term, weighing 2500g or more, and showing an adequate size for gestational age, Brazil, 2015-2018

Supplement 2.
Data Sharing Statement
