# Supplementary material for: All-Cause and Cause-Specific Mortality in Children With Congenital Zika Syndrome in Brazil
Source: JAMA Netw Open. 2025 Jan 23;8(1):e2456042. doi: 10.1001/jamanetworkopen.2024.56042 (PMC11758593; doi:10.1001/jamanetworkopen.2024.56042)
Supplement: Supplement 1. — eMethods. Criteria for defining a suspected case of Congenital Zika Syndrome (CZS), defined by the Brazilian Ministry of Health eReferences eTable. Baseline characteristics of live births included and excluded from the adjusted analysis, according to congenital Zika syndrome (CZS) Status, Brazil, 2015-2018 eFigure. All-cause and cause-specific mortality hazard ratio from respiratory, infectious and parasitic, and nervous system diseases, by age group, of 9,488,463 live births born at term, weighing 2500g or more, and showing an adequate size for gestational age, Brazil, 2015-2018 [file jamanetwopen-e2456042-s001.pdf]

Supplemental Online Content

Cardim LL, Costa MdCN, Rodrigues LC, et al. All-cause and cause-specific mortality in children with congenital Zika syndrome in Brazil. *JAMA Netw Open*. 2025;8(1):e2456042. doi:10.1001/jamanetworkopen.2024.56042

eMethods. Criteria for defining a suspected case of Congenital Zika Syndrome (CZS), defined by the Brazilian Ministry of Health
eReferences
eTable. Baseline characteristics of live births included and excluded from the adjusted analysis, according to congenital Zika syndrome (CZS) Status, Brazil, 2015-2018
eFigure. All-cause and cause-specific mortality hazard ratio from respiratory, infectious and parasitic, and nervous system diseases, by age group, of 9,488,463 live births born at term, weighing 2500g or more, and showing an adequate size for gestational age, Brazil, 2015-2018

This supplemental material has been provided by the authors to give readers additional information about their work.

© 2025 Cardim LL et al. *JAMA Network Open*.

eMethods. Criteria for defining a suspected case of Congenital Zika Syndrome (CZS), defined by the Brazilian Ministry of Health

Criteria for suspected case of CZS, defined by the Ministry of Health: 1) Cranial circumference less than 2 standard deviations, according to sex and gestational age, according to INTERGROWTH-21st growth curve;[1] 2) Craniofacial disproportion; 3) Arthrogryposis; 4) Ultrasound with altered pattern during pregnancy; 5) Persistence of two or more neurological, visual or auditory manifestations when there is no other known cause, regardless of maternal history; 6) Two or more neurological, visual or auditory manifestations, even non-persistent, whose mother presented a history of suspected or confirmed Zika virus infection during pregnancy; 7) Change in growth/neuropsychomotor development, without defined cause, with a history of suspected Zika virus infection during pregnancy; 8) Image exam with the presence of cerebral calcifications or ventricular alterations.[2]

eReferences

1. The Global Health Network. INTERGROWTH-21st. https://intergrowth21.tghn.org/about/sobre-intergrowth-21st/.
2. Brasil. Ministério da Saúde. Secretaria de Vigilância em Saúde. Secretaria de Atenção à Saúde. *Orientações Integradas de Vigilância e Atenção à Saúde No Âmbito Da Emergência de Saúde Pública de Importância Nacional: Procedimentos Para o Monitoramento Das Alterações No Crescimento e Desenvolvimento a Partir Da Gestação Até a Primeira Infância, Relac*. Ministério. Brasilia; 2017. https://portaldeboaspraticas.iff.fiocruz.br/wp-content/uploads/2019/02/orientacoes-integradas-vigilancia-atencao.pdf.

© 2025 Cardim LL et al. *JAMA Network Open*.

eTable. Baseline characteristics of live births included and excluded from the adjusted analysis, according to congenital Zika syndrome (CZS) Status, Brazil, 2015-2018.

Characteristics	Live Birth Included in the Adjusted Analysis		Live Birth Excluded in the Adjusted Analysis	
	Live Birth Without CZS	Live Birth With CZS	Live Birth Without CZS	Live Birth With CZS
	No (%)	No (%)	No (%)	No (%)
Maternal age (years)				
< 20	1819659 (17.0)	656 (23.3)	123749 (17.8)	56 (21.5)
20 - 34	7382142 (69.1)	1865 (66.1)	480511 (69.0)	169 (65.0)
≥ 35	1486435 (13.9)	299 (10.6)	91855 (13.2)	35 (13.5)
Missing	0 (0.0)	0 (0.0)	0 (0.0)	0 (0.0)
Maternal education (years)				
None	50502 (0.5)	17 (0.6)	3964 (0.8)	1 (0.5)
1 - 3	240516 (2.3)	89 (3.2)	21822 (4.1)	6 (2.8)
4 - 7	1811407 (17.0)	631 (22.4)	105964 (20.0)	48 (22.1)
≥ 8	8585811 (80.3)	2083 (73.9)	399308 (75.2)	162 (74.7)
Missing	0 (0.0)	0 (0.0)	165057 (23.7)	43 (16.5)
Maternal race/ethnicity				
Asian descendant	43580 (0.4)	11 (0.4)	679 (0.4)	0 (0.0)
Black	595465 (5.6)	189 (6.7)	8999 (4.9)	2 (3.2)
Indigenous	87620 (0.8)	18 (0.6)	5381 (2.9)	1 (1.6)
Pardo	6046985 (56.6)	2088 (74.0)	131072 (71.3)	49 (79.0)
White	3914586 (36.6)	514 (18.3)	37707 (20.5)	10 (16.1)
Missing	0 (0.0)	0 (0.0)	512277 (73.6)	198 (76.2)
Marital Status				
Single	4613149 (43.2)	1456 (51.6)	239794 (42.1)	101 (45.7)
Widow	18403 (0.2)	3 (0.1)	1134 (0.2)	2 (0.9)
Divorced	127185 (1.2)	24 (0.9)	5801 (1.0)	1 (0.5)
Married/union	5929499 (55.5)	1337 (47.4)	322247 (56.6)	117 (52.9)
Missing	0 (0.0)	0 (0.0)	127139 (18.3)	39 (15.0)
Year of birth				
2015	2724289 (25.5)	1096 (38.9)	199349 (28.6)	78 (30.0)
2016	2597716 (24.3)	1304 (46.2)	173192 (24.9)	132 (50.8)
2017	2664927 (24.9)	278 (9.9)	168128 (24.3)	31 (11.9)
2018	2701304 (25.3)	142 (5.0)	154446 (22.2)	19 (7.3)
Birth region				
Southeast	4317031 (40.4)	665 (23.6)	149698 (21.5)	42 (16.2)
North	1156831 (10.8)	163 (5.8)	71102 (10.2)	7 (2.7)
Northeast	2867314 (26.8)	1741 (61.7)	335877 (48.3)	174 (66.9)
South	1509595 (14.1)	40 (1.4)	32175 (4.6)	2 (0.8)
Central West	837465 (7.8)	211 (7.5)	107263 (15.4)	35 (13.5)
Missing	0 (0.0)	0 (0.0)	0 (0.0)	0 (0.0)

© 2025 Cardim LL et al. *JAMA Network Open.*

Sex of the newborn				
Female	5213475 (48.8)	1497 (53.1)	339097 (48.8)	133 (52.8)
Male	5474761 (51.2)	1323 (46.9)	356391 (51.2)	119 (47.2)
Missing	0 (0.0)	0 (0.0)	627 (0.1)	8 (3.1)

© 2025 Cardim LL et al. *JAMA Network Open*.

eFigure. All-cause and cause-specific mortality hazard ratio from respiratory, infectious and parasitic, and nervous system diseases, by age group, of 9,488,463 live births born at term, weighing 2500g or more, and showing an adequate size for gestational age, Brazil, 2015-2018.

Cause of Mortality	Crude Model HR (95% CI)
**Up to 5 years**	
All-cause	19.94 (16.83-23.63)
NSD	85.87 (63.09-116.88)
IPD	36.97 (28.29-48.30)
RSD	35.70 (27.58-46.22)
**4 to <5 years**	
All-cause	29.66 (14.80-59.47)
NSD	142.48 (52.52-386.52)
IPD	77.75 (28.90-209.22)
RSD	70.88 (26.37-190.57)
**3 to <4 years**	
All-cause	21.82 (10.90-43.70)
NSD	56.93 (18.24-177.73)
IPD	34.69 (11.14-108.00)
RSD	52.79 (21.87-127.46)
**2 to <3 years**	
All-cause	41.70 (27.15-64.05)
NSD	125.40 (62.27-252.53)
IPD	76.42 (40.96-142.58)
RSD	64.24 (34.45-119.78)
**1 to <2 years**	
All-cause	29.44 (19.88-43.61)
NSD	103.28 (58.40-182.65)
IPD	51.92 (31.24-86.28)
RSD	40.76 (24.10-68.93)
**0 to <1 year**	
All-cause	15.20 (12.06-19.16)
NSD	64.41(38.05-109.03)
IPD	24.33 (16.01-36.98)
RSD	25.63 (17.31-37.96)

A - Crude Model

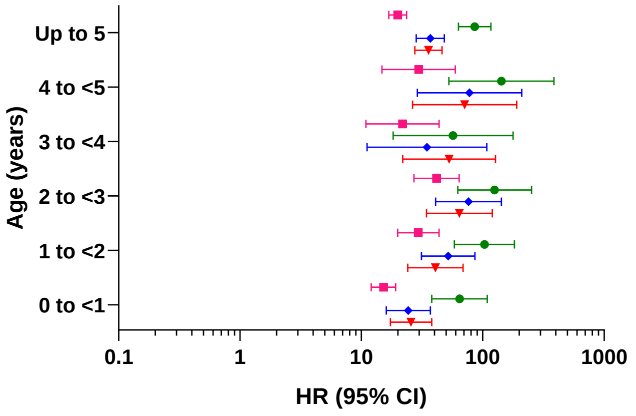

Cause of Mortality	Adjusted model* HR (95% CI)
**Up to 5 years**	
All-cause	16.94 (14.14-20.30)
NSD	69.77 (49.63-98.09)
IPD	29.64 (22.16-39.64)
RSD	28.03 (21.09-37.25)
**4 to <5 years**	
All-cause	23.98 (11.37-50.60)
NSD	113.64 (35.30-365.86)
IPD	84.49 (30.94-230.77)
RSD	50.61(16.03-159.83)
**3 to <4 years**	
All-cause	19.72 (9.37-41.50)
NSD	50.51(16.06-158.84)
IPD	21.84 (5.42-87.93)
RSD	40.34 (15.00-108.50)
**2 to <3 years**	
All-cause	43.74 (28.12-68.03)
NSD	105.74 (49.63-225.29)
IPD	65.16 (33.62-126.31)
RSD	52.61(27.18-101.82)
**1 to <2 years**	
All-cause	26.12 (17.16-39.75)
NSD	92.18 (50.53-168.13)
IPD	45.75 (26.44-79.15)
RSD	36.77 (21.27-63.57)
**0 to <1 year**	
All-cause	12.30 (9.59-15.79)
NSD	45.99 (24.62-85.91)
IPD	18.21(11.45-28.95)
RSD	18.44 (11.74-28.95)

B - Adjusted Model*

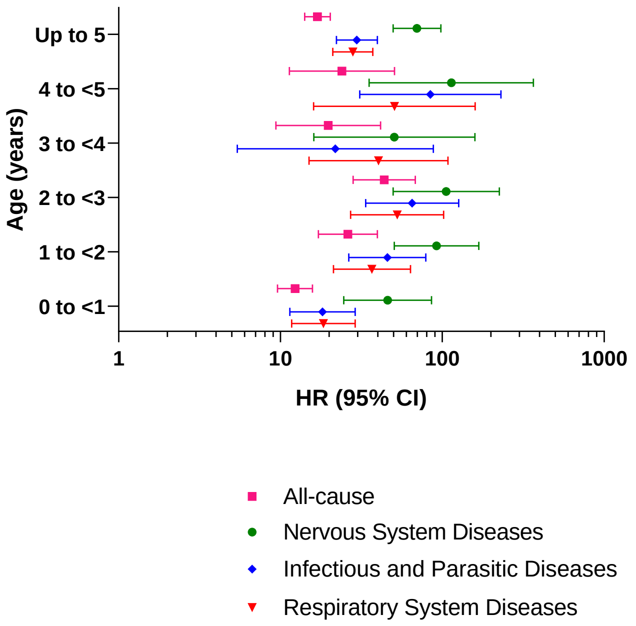

HR: Hazard Ratio
CI: Confidence Interval
NSD: Nervous System Disease
IPD: Infectious and Parasitic Disease
RSD: Respiratory System Disease
*Adusted by region, year of birth, maternal age, maternal education, maternal race/ethnicity, marital status and sex of the newborn

© 2025 Cardim LL et al. *JAMA Network Open*.
