# Supplementary material for: All-Cause and Cause-Specific Mortality in Children With Congenital Zika Syndrome in Brazil
Source: JAMA Netw Open. 2025 Jan 23;8(1):e2456042. doi: 10.1001/jamanetworkopen.2024.56042 (PMC11758593; doi:10.1001/jamanetworkopen.2024.56042)
Supplement: Supplement 2. — Data Sharing Statement [file jamanetwopen-e2456042-s002.pdf]

Data Sharing Statement

Cardim. All-Cause and Cause-Specific Mortality in Children With Congenital Zika Syndrome in Brazil. *JAMA Netw Open*. Published January 23, 2025.
doi:10.1001/jamanetworkopen.2024.56042

Data
Data available: No

Additional Information
Explanation for why data not available: The relevant data are available in the manuscript. Raw data are vailable upon reasonable request to the Center of Data and Knowledge Integration for Health (Centro de Integração de Dados e Conhecimentos para Saúde ¬-CIDACS) at Oswaldo Cruz Foundation (Fundação Oswaldo Cruz - FIOCRUZ). Any person who wishes to receive authorisation must: (1) be affiliated to CIDACS or be accepted as collaborators; (2) present a detailed research project together with approval by an appropriate Brazilian institutional research ethics committee; (3) provide a clear data plan restricted to the objectives of the proposed study and a summary of the analyses plan intended to guide the linkage and data extraction of the relevant set of records and variables; (4) sign terms of responsibility regarding the access and use of data; and (5) perform the analyses of datasets provided using the CIDACS data environment, a safe and secure infrastructure that provides remote access to de-identified or anonymised datasets and analysis tools. For more information: https://cidacs.bahia.fiocruz.br/
